# Epidemiology of Football Injuries of the German Bundesliga: A Media-Based, Prospective Analysis over 7 Consecutive Seasons

**DOI:** 10.1186/s40798-023-00563-x

**Published:** 2023-03-03

**Authors:** Karen aus der Fünten, Tobias Tröß, Abed Hadji, Florian Beaudouin, Ida Bo Steendahl, Tim Meyer

**Affiliations:** 1 Institute of Sports and Preventive Medicine, Saarland University, Campus, Geb. B. 8.2, 66123 Saarbrücken, Germany; 2 University Sports, Stuttgart University, Stuttgart, Germany

**Keywords:** Soccer, Sport injury, Injury patterns, Musculoskeletal system, Time loss

## Abstract

**Background:**

This study describes the implementation of a standardised, prospective injury database covering the entire 1st male German football league (“Bundesliga”) based on publicly available media data. For the first time, various media sources were used simultaneously as the external validity of media-generated data was low in the past compared to data obtained by way of the “gold standard”, i.e. by the teams’ medical staffs.

**Methods:**

The study covers 7 consecutive seasons (2014/15–2020/21). The primary data source was the online version of the sport-specific journal “kicker Sportmagazin™” complemented by further publicly available media data. Injury data collection followed the Fuller consensus statement on football injury studies.

**Results:**

During the 7 seasons, 6653 injuries occurred, thereof 3821 in training and 2832 in matches. The injury incidence rates (IRs) per 1000 football hours were 5.5 [95% CI 5.3–5.6], 25.9 [25.0–26.9] per 1000 match, and 3.4 [3.3–3.6] per 1000 training hours. Twenty-four per cent of the injuries (*n* = 1569, IR 1.3 [1.2–1.4]) affected the thigh, 15% (*n* = 1023, IR 0.8 [0.8–0.9]) the knee, and 13% (*n* = 856, IR 0.7 [0.7–0.8]) the ankle. Muscle/tendon injuries contributed 49% (*n* = 3288, IR 2.7 [2.6–2.8]), joint/ligament injuries 17% (*n* = 1152, IR 0.9 [0.9–1.0]), and contusions 13% (*n* = 855, IR 0.7 [0.7–0.8]). Compared to studies using injury reports from the clubs’ medical staff, media data revealed similar proportional distributions of the injuries, but the IRs tended towards the lower end. Obtaining specific locations or diagnosis especially with regard to minor injuries is difficult.

**Conclusions:**

Media data are convenient for investigating the quantity of injuries of an entire league, for identifying injuries for further subanalysis, and for analysing complex injuries. Future studies will focus on the identification of inter- and intraseasonal trends, players' individual injury histories, and risk factors for subsequent injuries. Furthermore, these data will be used in a complex system approach for developing a clinical decision support system, e.g. for return to play decisions.

## Key Points

- Publicly available media provides comprehensive injury data of the 1st German male football league (Bundesliga).
- Various media sources should be used simultaneously to obtain the injury data.
- Compared to studies using injury data from the clubs’ medical staff, media data revealed similar proportional distributions of the injuries, the IRs tended towards the lower end.

## Introduction

Injury rates in men’s professional football are considerably high. Overall injury incidence rates (IRs) vary between 4.8 and 14.4/1000 football hours, match IRs/1000 h between 22.7 and 43.5, and training IRs/1000 h between 2.8 and 11.2 [1–9]. Apart from a potential influence on the players’ (long-term) health, injury-related absences can impede team performance [10, 11]. Both aspects can lead to high economic costs for the clubs [12, 13]. Consequently, medical staff, sports scientists, and coaches alike are under constant pressure to keep their players healthy and injury free. Epidemiological studies are key for injury surveillance and prevention [14].

Injury studies so far have mainly included a selection of clubs within one or more leagues or have covered international tournaments. Rarely, they have covered an entire domestic league [1, 3, 4, 11, 12, 15–18]. The gold standard for obtaining injury data for research purposes is the collection via the clubs’ medical staffs. However, this approach has always been very difficult especially on an elite level as data are deemed strictly confidential and are not to be shared with external parties. Even this approach cannot guarantee data accuracy as incidence rates were reported to be significantly underestimated by about 20% according to Bjorneboe et al. [19].

In Germany, another option to obtain injury data is to use insurance records. All professional players are automatically insured by a statutory accident insurance. The clubs report football-related injuries to the insurance. However, insurance benefit can be claimed for acute injuries only, hence this database excludes overuse injuries. Additionally, only injuries lasting > 3 days are mandatory to be reported to the insurance [20]. Consequently, other methods of data gathering need to be considered especially if the aim is a comprehensive collection.

Using a media-based approach is an option. Over the last 10 years, there has been a massive increase in availability of such information. In Germany, several epidemiological studies covering injuries of the 1st male German football league (Bundesliga) have already been carried out. However, either these studies were retrospective in nature and/or based on one media source only, most often www.transfermarkt.com/de, or they did not contain the entire league and/or all season-related events, or they served to identify certain events within matches [5, 21–26]. Studies that used the webpage www.transfermarkt.de, as the only source for data collection underestimated the quantity of injuries by far. Hoenig et al. counted 6663 injuries within 10 Bundesliga-seasons (2009/10–2018/19), Leventer et al. [24] 3658 within six (2008/09–2013/14) [22], and Krutsch et al. [23] 171 for four teams within the 2015/16 season. The data collection of the latest and most comprehensive transfermarkt-study of Hoenig et al. [24] was performed retrospectively. One-third of the injuries could not be assigned to an injury location and almost another third (32%) not to an injury type.

Consequently, and for the first time, the present study utilised a prospective media-based approach based on various media sources and not just one. It followed a strict injury identification and verification protocol. It included all clubs of the 1st German male professional football league (Bundesliga) and covered 7 consecutive seasons. Data collection is ongoing.

The aims of this study were threefold:

1. To describe the implementation of a standardised, prospective injury registry covering the entire 1st male German football league (Bundesliga) based on various publicly available media data encompassing the seasons 2014/15 to 2020/21.
2. To compare the current results to previous studies covering Europe’s top leagues especially to those that used the gold standard of injury data collection, i.e. conducted prospectively by the clubs’ medical staff.
3. To outline advantages and disadvantages, limitations, and concessions of the media-based approach.

## Methods

### Study Design and Sample

A standardised, media-based, prospective analysis of football-related injuries was conducted in the 1st German male professional league (Bundesliga) encompassing the 2014/15 to 2020/21 seasons. The league includes 18 clubs. For study inclusion, players had to participate in at least one official seasonal match in a national or international competition. Their anthropometric data derived from the “kicker Sportmagazin™” journal. Neither research ethics board approval nor a trial registration was required as all data were collected from publicly available sources [25–27].

### Data Collection

Two researchers (sports scientists, TT for the 2014/15–2016/17 seasons, and AH for the seasons thereafter) recorded and categorised injury characteristics and their time loss according to the Fuller consensus statement for football injury research [15]. Data collection followed a standardised procedure by an a priori defined protocol and analysis plan. Injuries were primarily identified by a structured search in the online edition of the “kicker Sportmagazin™” [21, 25, 26]. It represents the leading football-specific magazine in Germany and is published twice weekly. The magazine’s journalists are in close, daily contact to the clubs. One journalist is responsible per club. After the initial identification, injuries were double checked and verified by further online sources such as web pages (ligainsider.de, transfermarkt.de), team homepages, TV sports channels (e.g. Sport 1), social media accounts of the teams or players (Twitter™, Facebook™, and Instagram™), clubs’ press conferences, and online available local newspapers. An injury had to pass through three main identification and verification steps to become acknowledged:

1. Identification of injuries in the “kicker Sportmagazin™” and registration of all given information in the database.
2. Confirmation of each injury by at least one further media source.
3. Supplementation of injury details via additional sources mentioned above.

All injuries were checked by one of the authors (orthopaedic surgeon, KadF) for medical plausibility.

### Match Exposure

Match exposure per team was calculated using the following calculation [15]: number of games × number of players on the field [11] × duration of the game in hours (1.5 h per match). The 36 match days in the “Bundesliga” season (34 regular games, 2 relegation games if applicable) as well as national (e.g. DFB-Pokal) and international cup games (e.g. Europa League, and the UEFA Champions League) were included in the analysis. Each season four German clubs played in the UEFA Champions League, two–four teams in the UEFA Europa League (two teams in the 2021/21 season, three in 2014/15, 2016/17, and 2018/19, and four in the remaining three seasons). Additionally, friendly games and extra time in knock-out games (2 × 15 min) were considered. Injuries that players sustained while representing their respective national team (single games as well as international tournaments) were included if they took place during the Bundesliga season and excluded outside this time frame (i.e. off-season in summer).

### Training Exposure

Training exposure per team was calculated by an estimation of an expert group with experience in the medical care of German professional players and according to previous publications [4, 21]. Additionally, two clubs of the “Bundesliga” provided precise information about their regular training schedules and the numbers of players present at each training session. Based on this, an average number of 20 players per training session was used for the calculation of training exposure. Training exposure included all supervised training sessions (team and individual training sessions as well as recovery sessions) conducted by the clubs’ coaches as well as the warm-up and cool-down on match days. Private training sessions led by external coaches were excluded. The season was divided into three phases: summer preparation (range 3–8 weeks, average 6.2 weeks), winter preparation (range 1–2 weeks, average 1.9 weeks), and the competition phase (range 34–45 weeks, average 40 weeks). Summer preparation comprises the restart of training after the season break in the summer until the first official match day (July/August). There is a winter break of 1.5–2 weeks duration that begins in the last week leading up to Christmas. The winter preparation stretches from the return to training after the winter break (end of December/beginning of January) to the first official match day after this break (January). The average weekly training exposure during the preparation phases was calculated with 15 h. The competition phase lasts from August–May/June. It is subdivided into weeks with one game per week (in this case 10 training hours per week) and two games per week (6.5 training hours per week).

### Injury Definition

The injury data were registered according to the Fuller consensus statement, which is based on the Orchard Sports Injury Classification System (OSICS). Injuries were defined as “any physical complaint sustained by a player that results from a football match or football training, irrespective of the need for medical attention or time loss from football activities” [15]. All injuries that occurred during friendly or regular matches were categorised as match injury. All injuries that occurred during a training session led by an official team coach or during warm-ups and cool-downs were categorised as training injury. If an injury enclosed more than one diagnosis, the most severe diagnosis was considered. The severities of injuries were categorised as minimal (time loss 1–3 days), mild (4–7 days), moderate (8–28 days), and severe (> 28 days) [15]. Injuries with a time loss < 1 day were excluded from the analysis as it is difficult to catch the 0-day time loss injuries and as it is common practice in research. If injuries outlived the end of the season players were followed until their recovery to determine the actual time loss.

### Concessions According to the Media-Based Approach

Only if the diagnosis or the description of the injury mechanism clearly implied a contact, it was classified as contact injury. This aspect mainly concerned training injuries as match injuries can be reviewed online, via telerecording, etc.

The following concessions had to be made to accommodate the media-generated data:

All adductor injuries were grouped to the hip/groin (and not the thigh) area. A differentiation between both sites was impossible.

If the media reported “muscular problems” that caused players to take a football break of ≥ 1 day, they were categorised as muscle injuries. Severe muscle injuries, i.e. lasting > 28 days that were described as “muscular problems”, “muscle injuries”, or “muscle strain”, were categorised as muscle tear.

For five aspects, additional categories were introduced. Three concerned the injury location and two the injury type. With regard to injury location, an often-used general term is “muscular problems”. For these injuries, we implemented the term “muscle injuries of the lower extremity including hip and groin unspecified” as especially injuries to the lower limbs will lead to a time loss in a football player. Furthermore, the locations “arm” and “unknown” were introduced, the latter if injuries could not be allocated to any body location. “Back problems” were often not specified any further referring to the specific spinal region and the injury type alike. That is why we used the general term “back” as an additional body region even though the majority assumingly affected the lumbar spine. As for the injury type, the term “muscle/tendon, bone/ligament back” was implemented to acknowledge that most of them, especially the less severe ones, were likely functional in nature, which often means some joint restrictions and muscle hypertonicity coexisting.

If ankle injuries lasted between 4 and 7 days (mild injuries) and the injury mechanism and/or description indicated an ankle sprain, injuries were classified as “suspected joint/ligament injuries” according to the likelihood of that injury even if this diagnosis was not specifically mentioned in the media. The same was true for knee injuries lasting 8–28 days (moderate injuries). Ankle injuries of ≥ 8 days (moderate and severe injuries) or severe knee injuries > 28 days duration (severe injuries) were categorised as ligament/joint injury along the same rationale as before.

If contact-related head injuries led to a time loss of ≥ 1d and if they had no indication of any accompanying injury, e.g. a facial fracture or a scalp wound, they were consequently counted as concussions and not as a “simple” head contusion.

### Statistical Analysis

All statistical analyses were performed using Statistica 28 (Statsoft Europe GmbH, Hamburg, Germany) and Excel Microsoft Office 365. Mean values were reported with standard deviations (± SD). Incidence rates (IRs) were calculated with the following formula:

$${\text{Incidence}} = \left( {{\text{number}}\;{\text{of}}\;{\text{injuries}}/{\text{hours}}\;{\text{of}}\;{\text{match}}\;{\text{or}}\;{\text{training or overall}}\;{\text{exposure}}} \right) \times 1000.$$

Confidence intervals (95% CI) were calculated as follows [28]:

$${\text{Lower }}95\% {\text{ CI}} = {\text{Incidence}}/e^{{1.96 \times ({\text{square}}\;{\text{root}}[1/{\text{number}}\;{\text{of}}\;{\text{incidents}}])}}$$

$${\text{Upper }}95\% {\text{ CI}} = {\text{Incidence}} * e^{{1.96 \times ({\text{square}}\;{\text{root}}[1/{\text{number}}\;{\text{of}}\;{\text{incidents}}])}}$$

Incidence rate ratios (IRRs) were used to compare match versus training injuries (match IR divided by training IR). The significance level was set at *p* < 0.05 for the α-error. The calculation of the injury burden followed the equation: total number of days absent × 1000/total exposure hours [29].

## Results

### Epidemiological Injury Data

The football exposure in 7 seasons totalled 1,220,223.5 h. The match exposure comprised 109,193.5 h and the training exposure 1,111,030 h. A total of 25 teams with an average squad size of 26 players (range 22–33) were included in the analysis. The mean age of the players was 25 ± 4 years, the mean height 183 ± 6 cm, the mean weight 78 ± 7 kg, and the mean BMI 23.28 ± 1.21 kg/m^2^.

### Injury Incidence Rates and Proportional Frequencies

**Fig. 1:**
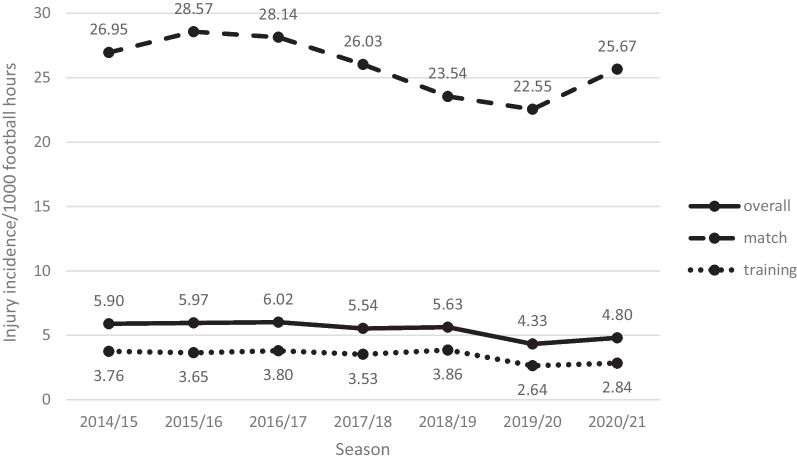
Time trends—injury incidence rates per 1000 football hours according to match, training, and the overall number of injuries

Within the 7 seasons, 6653 injuries occurred. The overall, match, and training injury incidence rates (IRs) are displayed in Table 1 and Fig. 1. The injury risk in matches was 7.5-fold higher compared to training sessions (incidence rate ratio IRR 7.5, 95% CI [7.2–7.9]). The mean number of injuries per season was 951 ± 112 (median 1004, range 787–1046). That means a team of 26 players experienced on average 52.8 ± 6.2 injuries (median 56, range 23–121) per season. 85 ± 5% of the players of each club suffered an injury within one season. The overall time loss amounted to 138,121 days (time loss per season in days: mean 19,733 ± 2198, median 19,248, range 17,949–24,016). The overall injury burden was 113 ± 11.3 absent days per 1000 football hours, 586 ± 24.3 days per 1000 match and 66.7 ± 10.9 days per 1000 training hours.

**Table 1:** Absolute numbers, proportions, IRs, proportions on overall time loss according to injury numbers, injury severity, and injury mechanism

	No. of injuries	Proportional frequency % [95% CI]	IR per 1000 football hours [95% CI]	% of days lost
All football injuries	6653	100	5.5 [5.3–5.6]	
Training injuries	2831	42.6 [41.4–43.8]	3.4 [3.3–3.6]	46.3
Match injuries	3822	57.4 [56.2–58.6]	25.9 [25.0–26.9]	53.7
*Injury severity*				
Severe (> 28 days)	1236	18.6 [17.6–19.5]	1.0 [1.0–1.1]	71.4
Moderate (7–28 days)	1869	28.1 [27.0–29.2]	1.5 [1.5–1.6]	20.4
Mild (4–7 days)	1370	20.6 [19.6–21.6]	1.1 [1.1–1.2]	5.2
Minimal (1–3 days)	2178	32.7 [31.6–33.9]	1.8 [1.7–1.9]	3.0
*Injury mechanism*				
Contact-related	2113	31.8 [30.6–32.9]	1.7 [1.7–1.8]	24.6
Non-contact	4540	68.2 [67.1–69.4]	3.7 [3.6–3.8]	75.4

*CI* confidence interval; *IR* injury incidence rate

Each club encountered a mean of 1096 ± 114 days injury-related “lost” days per season (median 940, range 250–2412 days). It took a player on average 21.0 ± 2.91 days (median 7, max 721 days) to return to competition. Most often, the injury severity was minimal (32%). Severe injuries contributed 19% (average time loss per injury 79.8 ± 70.5 days, median 55 days). Thirty-two per cent (*n* = 2113) of the injuries were identified as contact-related. They lasted on average 16.1 ± 35.0 days (median 4 days), non-contact injuries 23.0 ± 44.7 days (median 8 days) (Table 1).

Eighty-five per cent of all injuries affected the lower extremity. The three main injury locations were thigh (24%; time loss per injury in days: average 16.3 ± 23.9, median 8, max 240), knee (15%; time loss per injury in days: average 42.0 ± 75.0, median 10, max 721), and ankle (13%, time loss per injury in days: average 23.1 ± 43.8 median 7, max 476) (Table 2).

**Table 2:** Absolute numbers, proportions, IRs, proportions on overall time loss according to injury location

Injury location	No. of injuries	Proportional frequency % [95% CI] *n* = 6653 injuries	IR per 1000 football hours [95% CI]	% of days lost
Lower extremities	5622	84.5 [83.6–85.4]	4.6 [4.5–4.7]	88.5
Thigh	1569	23.6 [22.6–24.6]	1.3 [1.2–1.4]	18.5
Knee	1023	15.4 [14.5–16.2]	0.8 [0.8–0.9]	31.1
Ankle	856	12.8 [12.1–13.7]	0.7 [0.7–0.8]	14.3
Hip/groin	721	10.8 [10.1–11.6]	0.6 [0.6–0.6]	8.7
Lower leg/Achilles tendon	624	9.4 [8.7–10.4]	0.5 [0.5–0.6]	8.4
Foot	426	6.4 [5.8–7.0]	0.4 [0.3–0.4]	5.5
Lower extremity incl. hip/groin	403	6.0 [5.5–6.6]	0.3 [0.3–0.4]	1.9
Back (additional site)	317	4.8 [4.3–5.3]	0.3 [0.2–0.3]	2.2
Head	149	2.2 [1.9–2.6]	0.1 [0.1–0.1]	0.9
Shoulder/clavicle	119	1.8 [1.5–2.1]	0.1 [0.1–0.1]	2.4
Lower back/sacrum/pelvis	109	1.6 [1.3–1.9]	0.1 [01.-0.1]	2.6
Face	79	1.2 [0.9–1.5]	0.1 [0.1–0.1]	0.6
Chest/ribs/upper back	73	1.1 [0.9–1.4]	0.1 [0.1–0.1]	0.7
Neck/cervical spine	60	0.9 [0.7–1.1]	0.1 [0.0–0.1]	0.5
Hand/finger	42	0.6 [0.4–0.8]	0.0 [0.0–0.1]	0.5
Abdomen	40	0.6 [0.4–0.8]	0.0 [0.0–0.1]	0.4
Elbow	16	0.2 [0.1–0.4]	0.0 [0.0–0.0]	0.6
Wrist/forearm	5	0.1 [0.0–0.1]	0.0 [0.0–0.0]	0.04
Arm	3	0.1 [0.0–0.1]	0.0 [0.0–0.0]	0.02
Unknown	19	0.3 [0.2–0.4]	0.0 [0.0–0.0]	0.1

*CI* confidence interval; *IR* injury incidence rate

**Fig. 2:**
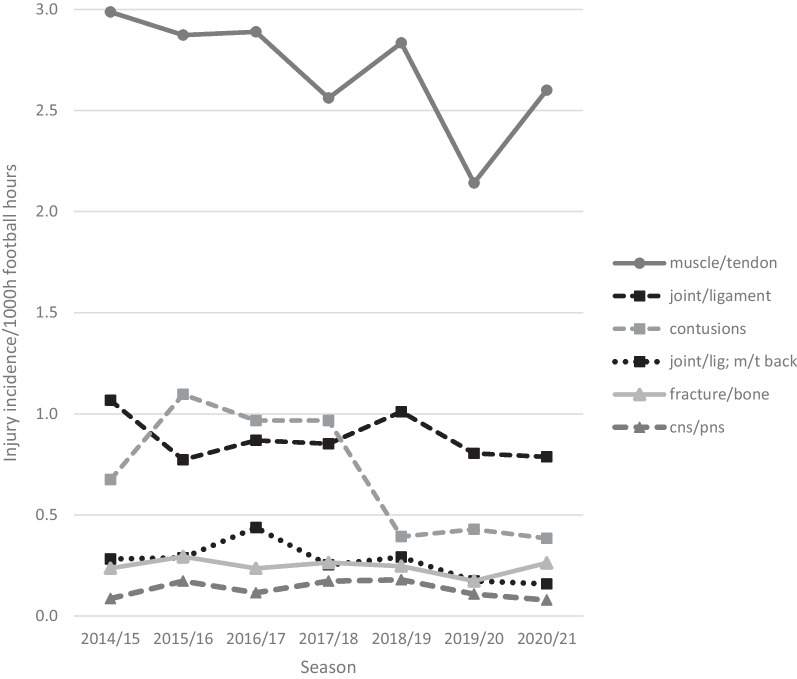
Time trends—injury incidence rates per 1000 football hours according to injury types. Lig ligament, M/t muscle/tendon, CNS/PNS central/peripheral nervous system

Muscle/tendon injuries contributed 49% (time loss per injury in days: average 15.6 ± 25.5, median 7, max 279), joint/ligament injuries 17% (suspected joint/ligament injuries included; time loss per injury in days: average 49.3 ± 73.3, median 23, max 721), and contusions 13% (time loss per injury in days: average 4.50 ± 5.06, median 3, max 65). Seven per cent of the injuries could not be allocated to any injury type and were thus classified as “unknown” (Table 3, Fig. 2).

**Table 3:** Absolute numbers, proportions, IRs, proportions on overall time loss according to injury types

Injury types	No. of injuries	Proportional frequency % [95% CI] *n* = 6653 injuries	IR per 1000 football hours [95% CI]	% of days lost
Muscle/tendon	3288	49.4 [48.2–50.6]	2.7 [2.6–2.8]	37.0
Joint/ligament	1075	16.2 [15.3–17.0]	0.9 [0.9–1.0]	41.1
Contusion	855	12.9 [12.1–13.7]	0.7 [0.7–0.8]	2.8
Muscle/tendon/joint/ligament (additional injury type back)	329	4.9 [4.4–5.5]	0.3 [0.2–0.3]	1.5
Fracture/bone	298	4.5 [4.0–5.0]	0.2 [0.2–0.3]	11.4
CNS/PNS	160	2.4 [2.0–2.8]	0.1 [0.1–0.2]	1.7
Laceration/skin injury	79	1.2 [0.9–1.5]	0.1 [0.1–0.1]	0.4
Joint/ligament suspected (additional injury type ankle, knee)	77	1.2 [0.9–1.4]	0.1 [0.1–0.1]	0.6
Other	15	0.2 [0.1–0.3]	0.0 [0.0–0.0]	0.6
Unknown	477	7.2 [6.6–7.8]	0.4 [0.4–0.4]	3.4

*CI* confidence interval; *IR* injury incidence rate; *CNS/PNS* central/peripheral nervous system

### Single Most Common Diagnoses

Table 4 holds the three injuries that are of major concern in football, i.e. thigh (non-contact) muscle, ankle, and knee ligament injuries (29%, *n* = 1957/6653). The total time loss for knee ligament injuries was 21,222 days in total, 77.7 ± 91.6 days on average with a median of 44 and a maximum of 518 days. The figures for the thigh (non-contact) muscle injuries were 23,657, 18.3 ± 25.3, 10, and 240 days, and for the ankle 11,389, 29.2 ± 37.6, 17, and 435 days. The same table displays the ten most frequently diagnosed injuries according to their absolute numbers (47%, *n* = 3130). The first three contributed 19% of all injuries (*n* = 1286, 11.8% of the overall time loss).

**Table 4:** Absolute numbers, proportions, IRs, proportions on overall time loss of the “Big 3” injuries and the “Top 10” diagnoses

Diagnosis	No. of injuries	Proportional frequency % [95% CI] *n* = 6653 injuries	IR per 1000 football hours [95% CI]	% of days lost
*3 main football injuries*				
Thigh muscle injuries (non-contact)	1294	19.5 [18.5–20.4]	1.1 [1.0–1.1]	17.1
Ankle ligament injuries	390	5.9 [5.3–6.4]	0.3 [0.3–0.4]	8.3
Knee ligament injuries	273	4.1 [3.6–4.6]	0.2 [0.2–0.3]	15.4
*10 most frequent diagnoses*				
Thigh muscle injury unspecified	537	8.1 [7.4–8.7]	0.4 [0.4–0.5]	2.2
Ankle sprain	390	5.9 [5.3–6.4]	0.3 [0.3–0.4]	8.3
Lower extremity incl. hip/groin muscle injury unspecified	359	5.4 [4.9–5.9]	0.3 [0.3–0.3]	1.4
Thigh muscle tear	348	5.2 [4.7–5.8]	0.3 [0.3–0.3]	8.5
Adductor muscle injury unspecified	317	4.8 [4.3–5.3]	0.3 [0.2–0.3]	1.5
Back problem	292	4.4 [3.9–4.9]	0.2 [0.2–0.3]	2.0
Thigh contusion	236	3.6 [3.1–4.0]	0.2 [0.2–0.2]	0.8
Ankle contusion	234	3.5 [3.1–4.0]	0.2 [0.2–0.2]	0.6
Knee injury unspecified	232	3.5 [3.1–3.9]	0.2 [0.2–0.2]	1.4
Foot contusion	186	2.8 [2.4–3.2]	0.2 [0.1–0.2]	0.6

*CI* confidence interval; *IR* injury incidence rate

Within the 7 seasons, there were 45 complete (IR 0.03 [0.03–0.04]/1000 football hours) and 5 partial anterior cruciate ligament ruptures (IR 0.004 [0.00–0.01]).

## Discussion

The simultaneous use of various media data as an alternative approach to provide comprehensive injury data of the German Bundesliga seems to be—in lack of a central injury registry based on the clubs’ medical records—a feasible option as media coverage of this league is high and obtained results seem plausible. Specific advantages and disadvantages of this approach are outlined at the end of the discussion.

A total of 6653 injuries occurred during seven consecutive seasons. That translates to an average of 53 injuries per season for a squad of 26 players, which represents the average squad size per club in the “Bundesliga”. This finding matches with the UEFA (Union of European Football Association) Elite Club Injury Study (ECIS). The authors reported 50 injuries per season for a squad of 25 players [30]. The latter study covered 1st league football clubs of 10 European countries participating in the UEFA Champions League, the highest club competition level in Europe. Bjorneboe et al. [6] who investigated the Norwegian male top league reported a maximum of 41 injuries per season and team. Norwegian clubs are less involved in international club competitions. Furthermore, season fixtures (14 teams instead of 18 in Germany; the Norwegian season runs from March–November versus August–May with a winter break in December/January of 3 weeks) and surfaces differ (Norway uses artificial turf and grass). All three aspects could explain differences. The latter explanations would not hold for the 1st French league though. French teams sustained on average only 37.2–41.2 injuries per season [31].

### Overall, Match, and Training Injuries

The overall, match, and training IRs are at the lower end compared to other European leagues (Table 5).

**Table 5:** (Inter)national comparison of overall, match, and training IRs

References	League(s), Nr. of team(s) of the respective league	Season(s), *n* = number of included seasons	Overall IR per 1000 football hours	Match IR per 1000 match hours	Training IR per 1000 training hours
Current study media-based	1st German 18/18	2014/15–2020/21 *n* = 7	5.5	25.9	3.4
Bjorneboe, Bahr [6]	1st Norwegian 14/14	2002–2007 *n* = 6	4.8	Not provided	Not provided
Noya Salces, Gomez-Carmona [9]	1st Spanish 16/20	2008/09 *n* = 1	5.7	43.5	3.6
Stubbe, van Beijsterveldt [3]	1st Dutch 8/18	2009/10 *n* = 1	6.2	32.8	2.8
Hagglund, Walden [32]	1st Swedish 11/14	2005 *n* = 1	7.7	28.1	4.7
Hagglund, Walden [8]	1st Swedish 12/14	2001–2002 *n* = 2	7.1/8.2	22.7/25.9	5.1/5.3
Hagglund, Walden [7]	1st Danish 8/12	January–June 2001 2nd leg of one season	14.4	28.2	11.2
Orhant, Chapellier [31]	1st French 20/20, 16 teams participating in both seasons	2018/19, 2020/21 *n* = 2	Not provided	24.4/29.4	Not provided
Lopez-Valenciano, Ruiz-Perez [33]	1st French, Spanish, Italian, English, and German (pooled data)	Systematic review and meta-analysis, studies until February 2018 included	7.6	35.5	3.6
Ekstrand, Spreco [34] UEFA-ECIS	UEFA Champions League (the best 1st league clubs of 10 European countries)	2001/02–2018/19 *n* = 18	6.6	23.8	3.4
aus der Fünten, Faude [4]	1st and 2nd German 5/18 1st, 2/18 2nd league	2008/09–2009/10 2nd legs of two seasons	5.9/6.6	26.5/31.5	2.7/4.0
Faude, Meyer [5] media-based	1st German 18/18	2005/06 *n* = 1	Not provided	37.5	Not provided

*IR* injury incidence rate; *ECIS* Elite Club Injury Study of the Union of European Football Associations (UEFA)

In contrast to the UEFA-ECIS [34], the present analysis included 66.6% of teams, which did not play internationally on the club level. This led to a higher training exposure as nationally playing teams almost always participated in one game only per week. A higher training exposure lowers the overall IR because the training incidence is substantially lower compared to the match IR. Ninety-one per cent of the overall exposure was attributed to training hours, whereas other studies described a proportion of 84–89% only [1, 3, 35]. It has to be considered that the current study had to estimate training exposure for obvious methodological reasons in contrast to all other studies.

Some of the studies quoted before include data from the 1st decade of the twenty-first century, whereas the present study only started in the 2014/15 season. The awareness of the importance of preventative measures has been ever increasing in the last 2 decades. However, such development cannot fully explain the observed phenomenon. Additional risk factors have been identified such as the leadership style of the head coach or an insufficient within team communication. Addressing those might have also contributed to a lower number of injuries in recent years [34].

Furthermore, the present study included the “Covid-19 season” 2019/20, which was interrupted for 2 months. This season displayed the lowest incidence rates of all seasons.

Insufficient media coverage of the injuries could also explain the lower numbers compared to studies using the gold standard for obtaining the injury data. However, compared to national data that stemmed from the second legs of the 2008/09 and 2009/10 seasons and that were retrieved by way of the gold standard IRs were quite similar (see Table 5) [4]. Whether these data truly represented the entire league(s) is debatable as only 7 out of 38 eligible teams of the 1st (*n* = 5) and 2nd (*n* = 2) professional male German football leagues took part. Furthermore, the length of the winter break that preceded the data acquisition period changed from 6.5 to 3.5 weeks between the included seasons. Ever since the winter break has remained with about 3 weeks.

The very first epidemiological analysis in German professional football was performed retrospectively but media-based, too by Faude et al. [5]. It covered the entire league [18 teams] of the 2004/05 season, match injuries only. The match IR was 37.5 and thus, considerably higher compared to the match IR of 25.9 of the present study. Faude et al. did not include friendly games, which are by far less competitive. German teams tend to play up to 20 “friendlies” per season. What is more, there are usually no restrictions on the number of replacements and there is no set match duration.

### Injury Types

Table 6 presents percentages and IRs of the three main injury types of various European leagues.

**Table 6:** (Inter)national comparison of injury type

References	League(s), Nr. of team(s) of the respective league	Season(s), *n* = number of included seasons	Muscle/tendon injuries % and IR per 1000 football hours	Joint/ligament injuries % and IR per 1000 football hours	Contusion injuries % and IR per 1000 football hours
Current study media-based	1st German 18/18	2014/15–2020/21 *n* = 7	49 2.7	16 0.9	13 0.7
Bjorneboe, Bahr [6]	1st Norwegian 14/14	2002–2007 *n* = 6	45 2.1	27 1.3	14 0.7
Noya Salces, Gomez-Carmona [9]	1st Spanish 20/20	2008/09 *n* = 1	54 3.0	24 1.4	14 0.8
Stubbe, van Beijsterveldt [3]	1st Dutch 8/18	2009/10 *n* = 1	39 2.3	19 1.2	18 1.1
Hagglund, Walden [32]	1st Swedish 11/14	2005 *n* = 1	44 3.4	19 1.5	18 1.4
Hagglund, Walden [8]	1st Swedish 12/14	2001 and 2002 *n* = 2	22 1.7	16 1.2	15 1.2
Hagglund, Walden [7]	1st Danish 8/12	January–June 2001 2nd leg of one season	21 3.0	23 3.3	14 2.1
Ekstrand, Hagglund [1] UEFA-ECIS	UEFA Champions League (the best 1st league clubs of 10 European countries)	2001/02–2008/09 *n* = 8	43 3.4	26 2.1	17 1.3
aus der Fünten, Faude [4]	1st and 2nd German, 5/18 1st, 2/18 2nd league	2008/09–2009/10 2nd legs of two seasons	42/34 2.5/2.2	27/30 1.6/1.9	19/17 1.1/1.1

*IR* injury incidence rate; *ECIS* Elite Club Injury Study of the Union of European Football Associations (UEFA)

In general, the percentages and IRs of muscle/tendon injuries display a high variance amongst studies with the media data being at the upper percentage end and somewhere in the middle of the IR range. This is most likely attributed to the high number of “muscular problem” injuries (*n* = 1471). Because they only lasted a few days, 47% less than 4 days, there is a chance that those injuries will not be reported to the full extent to a central injury registry as they might not be considered as injuries as such. Joint/ligament injuries seem to be underrepresented if media sources are used. Contusion injuries are at the lower end. The latter could be explained by the fact that an injury was only classified as contusion either if the media explicitly used this term or if the injury description strongly implied this injury type, e.g. a player was hitting the goal post with his foot and was able to play again after 3 days. The proportion of contact-related injuries overall does not seem to be underrepresented if media data is used as the current study contained 32%. A previous German study reported only 18% of such injuries [4], Hagglund et al. reported 35% within the 1st Swedish [32], and Stubbe et al. [3] 39% within the 1st Dutch male league. There seems to be quite a variety regarding the percentage of contact-related injuries with the media-based data sitting roughly in the middle (Tables 7 and 8).

**Table 7:** (Inter)national comparison of injury location

References	League(s), Nr. of team(s) of the respective league	Season(s) *n* = number of included seasons	Thigh injuries % and IR per 1000 football hours	Knee injuries % and IR per 1000 football hours	Ankle injuries% and IR per 1000 football hours	Hip/groin injuries% and IR per 1000 football hours	Lower leg/Achilles tendon injuries % and IR per 1000 football hours
Current study media-based	1st German 18/18	2014/15–2020/21 *n* = 7	24 1.3	15 0.8	13 0.7	11 0.6	9 0.5
Bjorneboe, Bahr [6]	1st Norwegian 14/14	2002–2007 *n* = 6	21 1.0	16 0.8	17 0.8	7 0.3	9 0.4
Noya Salces, Gomez-Carmona [9]	1st Spanish 16/20	2008/09 *n* = 1	37 2.1	11 0.6	14 0.8	14 0.8	10 0.6
Stubbe, van Beijsterveldt [3]	1st Dutch 8/18	2009/10 *n* = 1	23 1.4	21 1.3	11 0.7	11 0.7	12 0.8
Hagglund, Walden [32]	1st Swedish 11/14	2005 *n* = 1	23 1.7	16 1.2	14 1.0	18 1.4	10 0.8
Hagglund, Walden [8]	1st Swedish 12/14	2001 and 2002 *n* = 2	23 1.7	17 1.3	10 0.7	17 1.3	13 1.0
Hagglund, Walden [7]	1st Danish 8/12	January–June 2001 2nd leg of one season	22 3.2	21 3.0	13 1.9	15 2.1	11 1.7
Ekstrand, Hagglund [1] UEFA-ECIS	UEFA Champions League (the best 1st league clubs of 10 European countries)	2001/02–2008/09 *n* = 8	24 1.9	18 1.5	14 1.1	14 1.1	11 0.9
aus der Fünten, Faude [4]	1st and 2nd German 5/18 1st, 2/18 2nd league	2008/09–2009/10 2nd legs of two seasons	26/26 2.0/1.7	17/25 1.0/1.6	17/16 1.0/1.1	3/5 0.2/0.3	9/7 0.6/0.6

*IR* injury incidence rate. ECIS Elite Club Injury Study of the Union of European Football Associations (UEFA)

**Table 8:** (Inter)national comparison of injury severity

References	League(s), Nr. of team(s) of the respective league	Season(s), *n* = number of included seasons	Severe injuries % and IR per 1000 football hours	Moderate injuries % and IR per 1000 football hours	Mild injuries % and IR per 1000 football hours	Minimal injuries % and IR per 1000 football hours
Current study media-based	1st German 18/18	2014/15–2020/21 *n* = 7	19 1.0	28 1.5	21 1.1	33 1.8
Bjorneboe, Bahr [6]	1st Norwegian 14/14	2002–2007 *n* = 6	21 1.0 Time loss > 21 days!	28 1.3 Time loss 8–21 days!	51 2.4 Time loss 1–7 days!	See mild injuries
Noya Salces, Gomez-Carmona [36]	1st Spanish 16/20	2008/09 *n* = 1	8 0.5	29 1.7	27 1.5	36 2.0
Stubbe, van Beijsterveldt [3]	1st Dutch 8/18	2009/10 *n* = 1	12 1.0	24 2.1	32 2.0	18 1.0
Hagglund, Walden [32]	1st Swedish 11/14	2005 *n* = 1	9 0.7	26 2.0	29 2.2	36 2.8
Hagglund, Walden [8]	1st Swedish 12/14	2001 and 2002 *n* = 2	11 0.9	37 2.8	28 2.1	33 2.6
Hagglund, Walden [7]	1st Danish 8/12	January–June 2001 2nd leg of one season	12 1.7	21 3.0	24 3.5	43 6.2
Ekstrand, Hagglund [1] UEFA-ECIS	UEFA Champions League (the best 1st league clubs of 10 European countries)	2001/02–2008/09 *n* = 8	16 1.3	Not provided	Not provided	Not provided
aus der Fünten, Faude [4]	1st and 2nd German (5/18 1st, 2/18 2nd league)	2008/09–2009/10 2nd legs of two seasons	13/18 0.8/1.2	29/34 1.7/2.2	29/19 1.7/1.2	29/30 1.7/2.0

Time losses per injury severity unless stated otherwise [days]: slight 1–3, light 4–7, moderate 8–28, severe > 28

*IR* injury incidence rate; *ECIS* Elite Club Injury Study of the Union of European Football Associations (UEFA)

### Injury Location

The proportional distribution of the injury location of the media-based study matches other studies. The IRs tend again towards the lower end of the spectrum. Figures of the Norwegian league were quite similar [6].

Figures can vary substantially within a few seasons, between and within clubs [20]. Pooled data from Lopez-Valenciano et al. [33] described a thigh IR of 1.8, a knee IR of 1.2, an ankle IR of 1.1, a hip/groin IR of 0.9, and a lower leg IR of 0.8. They included leagues from all over the world and international tournaments, too. The latter are associated with substantially higher IRs. Junge et al. [16] investigated the World Cups 1998–2012. The match IR was 77.3. Apart from the reasons mentioned before, 6.1% of the media-based injuries (IR 0.33) could only be allocated to the lower limbs but not to a specific region. That might explain some of the observed effects.

### Injury Severity

Severe injuries are well covered in the media and are therefore most likely overrepresented [23].

However, compared to the “gold standard” German data, the recent proportion of 19% severe injuries and the IR of 1.0 was not far off. The same is true for all other subcategories.

The media data do not seem to underestimate the (proportion of) less severe injuries, i.e. lasting ≤ 7 days, as they constituted 53% (IR 2.9).

The injury burden (absent days per 1000 exposure hours) in the 18-year UEFA-ECIS was 61 for training and 505 for matches [34]. The current data resulted in 67 and 586 days, respectively, and were quite similar.

On the whole, it is difficult to compare data of various studies as they vary, e.g. with regard to the number of included clubs (several vs. multiple vs. all teams of one league) or seasons (one—18 seasons), the level of play (national vs. international vs. both), the number of considered games (with or without national team and/or friendly games), the climate, the playing surfaces, the injury definition (some studies included injuries only if they last ≥ 72 h), and the method of data collection, centralised versus decentralised the latter increasing the likelihood of more complete data reporting as it should be in the clubs’ vested interest to capture their data as accurate and complete as possible. Furthermore, even within one league there can be substantial interseasonal variance. The current study revealed similar proportions regarding injury characteristics compared to other European studies, whereas the IRs tended towards the lower end of the spectrum. Possible explanations were provided above. Data from the German league that were obtained via the gold standard were quite well matched.

### General Advantages and Disadvantages, Limitations, and Concessions of this Media-Based Approach

#### Advantages

The gold standard of injury data collection is acquisition and transmission by the clubs’ medical staff. The media data used in this study were publicly available. Thus, the clubs are not personally involved. This takes out the clubs’ two main concerns why they do not like to and why they consequently do not partake in (scientific) studies: the possible breach of data confidentiality as well as the additional time expenditure.

The internal validity of this media-based study is likely high due to a strict data collection protocol and a high consistency amongst researchers involved.

The media-based approach allows for inter- and intraseasonal comparisons of injury characteristics, time loss, and time trends. The media data have already proven to be useful for a couple of aspects such as the identification of injuries, e.g. for further analysing injury mechanisms via video material [25, 26], or a more detailed analysis of severe injuries [21]. Multiple injuries sustained at the same time can be investigated further as often not just the diagnosis of the most severe injury is described but also accompanying (minor) injuries. The latter is important, e.g. in the light of head injuries. Players with facial fractures are likely to be categorised as bony injury as the major one and a possible accompanying concussion goes unrecognised.

#### Disadvantages/Limitations/Concessions

Media coverage for a media-based study must be high. A professional league/event and a well-recognised sport in the respective country are indispensable prerequisites. The higher the popularity of a club or player the higher the chances of more detailed information. To gain as much information as possible, this study used various media sources simultaneously for the injuries’ identification and verification process. This approach is quite time-consuming. However, even with this approach and with high level clubs some injuries, pathologies or rehabilitation processes do not transcend directly or clearly to the media. That means that not all injuries become known. Medical confidentiality as well as players’ privacy might well play a role here.

If various media sources are used it has to be considered that they might rely on each other, which can lead to misunderstandings and errors. In case the media provided the name of their source of information, this source was not used for the verification process.

The primary injury identification was made through the kicker Sportmagazin™ with one reporter being responsible for each club. That means that the initial capturing of an injury is highly person dependent, which can lead to a sampling bias. Generally, the less severe the injury, the higher the probability to encounter limitations.

Training exposure had to be estimated based on experts’ opinion as only 2 out of 18 Bundesliga clubs were willing to share their data. This refers to the number of attending players and to the duration of the training sessions alike. That can lead to an over- as well as to an underestimation of the training and to an inverse effect on the overall injury incidence.

With regard to the injury location, it has to be considered that the implemented regions “back” and “arm” constituted 5% (*n* = 317/6653) and 0.01% (*n* = 3), respectively. 0.3% (*n* = 19/6653) of the injuries could not be allocated to any body location. That also decreases data accuracy.

A total of 477 injuries (7%; 3% of the total time loss; min 50, max 89 injuries per season) could not be assigned to a specific injury type. The implemented terms “muscle injuries of the lower extremity including hip and groin unspecified” and “muscle/tendon, bone/ligament back” accounted for 5% each (*n* = 359 and 329 out of 6653 injuries). Attributing thigh muscle injuries to the quadriceps and hamstring group was possible in less than 25%. On very rare occasions, the specific muscle within these muscle groups was named. The “suspected joint/ligament injury category” comprised 1% of all injuries (*n* = 76/6653). This problem could only be solved if specific diagnoses would be made available by the clubs. Over the years, the amount and detail of media data on football injuries has increased massively. A further increase in the future is expected. However, that does not mean that data accuracy is improving at the same time.

Diagnoses provided by the media might not be accurate as they are transmitted by lay persons. If players were out for at least one day due to “muscular problems” it was counted as an injury. In this case, it might not have been a true injury in the medical sense but only a precautionary measure. This aspect can lead to an overestimation of the number of muscle injuries. However, it does not seem the case as the percentage of muscle injuries was comparable, and their injury incidence was at the lower end to studies compared to studies obtaining injury data by way of gold standard.

If players had to take ≥ 1 day out from a contact-related head injury, it was classified as a concussion. This could lead to an overestimation with regard to the frequency of concussions. The problem is that there is no agreement how long signs and symptoms have to last, and how severe they have to be at any given time, to call it a concussion. The more conservative the approach the higher the number and subsequently the injury incidence of concussions will be.

## Conclusions

The need to collect injury data from media arose from the very limited accessibility of clubs’ injury data. The results of this study indicate that media data are generally very comprehensive and seem plausible if a strict injury identification and verification protocol is in place. Various media sources should be used simultaneously as injury data are much closer to investigations that are fortunate enough to utilise the gold standard data compared data deriving from a single source. Due to the strict data protocol, the internal validity is likely high.

The more severe the injury, the better the accuracy of the injury data. Obtaining a specific diagnosis of minor injuries is very challenging and not always possible. Proportional injury distributions are comparable to studies using the gold standard of data collection, whereas IRs tend towards the lower end. Thus, these data should be interpreted with caution.

## Data Availability

The datasets used and analysed during the current study are available from the corresponding author on reasonable request.

## Abbreviations

CI: Confidence interval
CNS/PNS: Central/peripheral nervous system
e.g.: For example
i.e.: That is
IR: Injury incidence rate
Lig: Ligament
m/t: Muscle/tendon
TM: Trademark
ECIS: Elite Club Injury Study of the Union of European Football Associations (UEFA)
